# Relationship between the benefits of paraspinal mapping and diffusion tensor imaging and the increase of decompression levels determined by conventional magnetic resonance imaging in degenerative lumbar spinal stenosis

**DOI:** 10.1186/s13018-019-1065-5

**Published:** 2019-01-22

**Authors:** Hua-Biao Chen, Min Chen, Hong-Hui Peng, Qi-Feng Xu, Xin-Chun Li, Bo Bai

**Affiliations:** 1 Departments of Spine Surgery, Affiliated Huizhou Central People Hospital, Sun Yat-Sen University and Guangdong Medical University, 41Goose Ridge North Road, 516001 Huizhou City, Guangdong Province People’s Republic of China; 2 Guangdong Key Laboratory of Orthopaedic Technology and Implant Materials, First Affiliated Hospital, Guangzhou Medical University, 151Yanjiang Road, 510120 Guangzhou, People’s Republic of China; 3 Department of Orthopaedic, First Affiliated Hospital, Guangzhou Medical University, 151Yanjiang Road, 510120 Guangzhou, People’s Republic of China; 4 Department of Radiology, First Affiliated Hospital, Guangzhou Medical University, 151Yanjiang Road, 510120 Guangzhou, People’s Republic of China; 5 Department of Electromyography, First Affiliated Hospital, Guangzhou Medical University, 151Yanjiang Road, 510120 Guangzhou, People’s Republic of China

**Keywords:** Diffuse tensor imagining, Lumbar spinal stenosis, Paraspinal mapping

## Abstract

**Background:**

In lumbar spinal stenosis (LSS), at most times, several levels are impaired and selecting the correct level remains a common problem for surgeons, as surgery remains invasive, and extended laminectomy may lead to secondary surgical complications. Therefore, helping to select the correct level may be useful for surgeons. The use of diffuse tensor imaging (DTI) and paraspinal mapping (PM) in addition to conventional magnetic resonance imaging (MRI) may be helpful (Chen et al., J Orthop Surg Res 11:47, 2016). However, with decompression levels determined by conventional magnetic resonance imaging (MRI) increasing, whether the benefits of reducing decompression level of conventional MRI + (DTI or PM) will be more obvious is unknown.

**Methods:**

Reduced surgical levels that were different between levels determined by conventional MRI + (DTI or PM) and conventional MRI + neurogenic examination (NE) between groups were compared. Treatment outcome measures were performed at 2 weeks, 3 months, 6 months, and 12 months postoperatively.

**Results:**

The reduced levels of three groups showed no statistically significant differences between each other except for two levels and four levels (two levels/three levels, *p* = 0.085; two levels/four levels, *p* = 0.039; three levels/ four levels, *p* = 0.506, respectively).

**Conclusions:**

With surgical levels determined by conventional MRI increasing, the benefits of DTI and PM will be uncertainly more obvious.

## Introduction

The term lumbar spinal stenosis (LSS) is commonly used to describe patients with symptoms related to anatomical reduction in lumbar spinal canal. Among older individuals, LSS is a highly disabling condition [1] and is the most common reason for spinal surgery [2, 3].The most common procedure involves a decompressive laminectomy of the structures thought to cause nerve root irritation.

The challenge for anatomically based determination is that, while necessary for the diagnosis of LSS, it is not sufficient to determine the severity of symptoms which lead a patient to seek treatment [4]. The extent of narrowing of the spinal canal correlates poorly with symptom severity, and radiologically significant lumbar spinal stenosis is also found in asymptomatic individuals [4–7]. As a consequence, correlating symptoms and physical examination findings with the decompression levels based on common imaging results are not reliable. In patients who have no concordance between radiological and clinical symptoms, the surgical levels determined by conventional magnetic resonance imaging (conventional MRI) and neurogenic examination (NE) may lead to a more extensive surgery and secondary surgical complications. It is important to select the correct level in clinical practice.

The use of diffuse tensor imaging (DTI) and paraspinal mapping (PM) techniques can reduce the decompression levels of lumbar spinal stenosis than conventional MRI + NE [8]. However, with decompression levels determined by conventional MRI + NE increasing, whether the benefits of conventional MRI + (DTI or PM) will be more obvious is unknown.

## Materials and methods

### Enrollment and grouping

Symptomatic patients of aged 40–90 years with degenerative lumbar spinal stenosis detected on conventional MRI or radiography from October 2015 to October 2017 were enrolled in this study. Since stenosis defining features can be seen on conventional MRI before and more clearer than changes consistent with stenosis can be detected on radiography, patients with degenerative lumbar spinal stenosis on conventional MRI were eligible. The inclusion criteria were as follows: patients had neuro claudication including both lower back pain and one leg pain that was consistent with a lumbar spinal stenosis and had persisted for at least 6 months despite pharmacologic treatment, physical therapy, or limitation of activity. Leg pain was defined as the pain below the buttocks [9]. Neurogenic claudication was typical with severe pain or/and disability, and a pronounced constriction of the lumbar spinal canal, therefore, was considered for decompression treatment [10]. NE was performed by an experienced spine surgeon, who was blind to the treatment of the patient. Levels of decompression determined by conventional MRI were ≥ 2. Patients were excluded if they had history of heavy alcohol consumption, history of lower back surgery [11, 12], evidence of polyneuropathy, or technically inadequate conventional MRI or electromyography (EMG) results.

In terms of the decompression levels determined by conventional MRI, the patients were divided into three groups: 1, two levels; 2, three levels; and 3, four levels.

### Interventions

All patients underwent conventional MRI and symptoms, signs, X-ray, DTI, and PM examinations and went for decompression surgeries (laminectomies or laminotomies) with surgical levels determined by conventional MRI + (PM or DTI). All surgeons were trained and performed at least for 50 lumbar spinal decompression surgeries annually. PM and DTI were described as below.

### Paraspinal mapping (MiniPM)

**Fig. 1:**
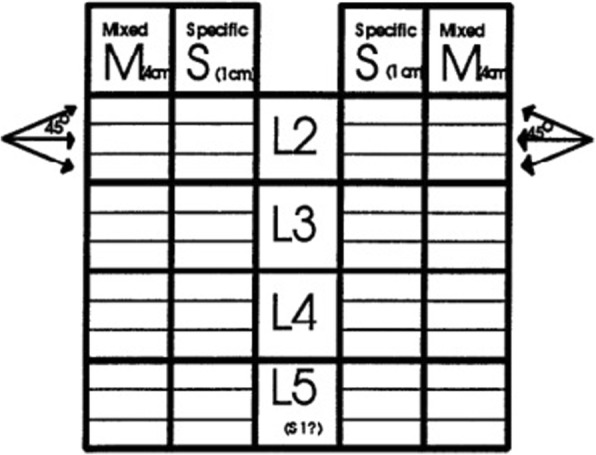
The scoresheet: spontaneous activity is scored separately for the first 4 cm insertion (placed in the scoresheet M column) and in the last l cm insertion (placed in the scoresheet S column) [22]. In this study, L1 and S1 nerve roots were added to the scoresheet, PM score was the summary of all plus at one level nerve root, one side

**Fig. 2:**
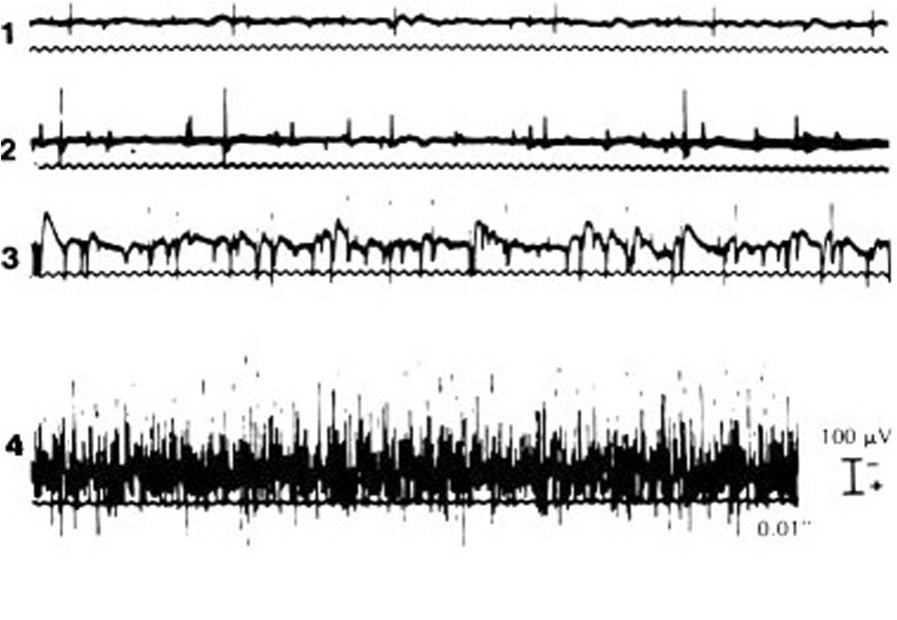
Fibrillation potentials in denervated muscle. Grades of activity: 1+, fibrillation potentials persistent in at least two areas; 2+, moderate number of persistent fibrillation potentials in three or more areas; 3+, large number of persistent discharges in all areas; 4+, profuse, widespread, persistent discharges that fill the baseline [23]

Typically, the total MiniPM score was used to indicate the extent of paraspinal denervation. In this study, the MiniPM score at each nerve root, a summary of six scores (only a summary of three scores of the 5 needle point-S1 nerve root, represented the score) were shown to be associated with the level of the radiculopathy used [13] (Fig. 1). Denervation appeared if the paraspinal muscles showed fibrillation potentials, positive sharp waves, or complex repetitive discharges [14] (Fig. 2). The standard was as follows: if a summary of six scores of one level (only a summary of three scores of the fifth needle point-S1 nerve root, represented the score.) was ≥ 2 one side, the level should be treated surgically. The PM examination was performed by a qualified electro-diagnostic physician who was blind to the treatment of patients.

### Conventional MRI protocol

A 3.0-T conventional MRI scanner (Achiva; Philips, Netherlands) was used in this study. Sagittal T1-weighted fast spin-echo sequences were obtained using a 453/8.0 ms for TR/TE, 4/0.4-mm section thickness/gap; 176 × 290 matrix; 0.91 × 1.00 × 4.00-mm^3^ actual voxel size; 0.50 × 0.50 × 4.00 mm^3^ calculated voxel size and sagittal T2-weighted fast spin-echo (TR/TE, 3604/110) sequences were obtained using a 4/0.4-mm section thickness/gap; 176 × 290 matrix; 0.91 × 1.00 × 4.00-mm^3^ actual voxel size and 0.50 × 0.50 × 4.00 mm^3^ calculated voxel size.

The quantitative criteria used for central anatomical LSS were as follows: The dural sac cross-sectional area (DSCSA) ≥ 100 mm^2^ was considered normal, 76 to 100 mm^2^ was considered to be moderately stenotic, and ≤ 76 mm^2^ was classified as severely stenotic. The nerve root compromise in lateral recess was graded as follows: grade 0, no contact of the disc with the nerve root; grade 1, contact without deviation; grade 2, nerve root deviation; and grade 3, nerve root compression. A nerve root compression was considered to be present when the root was deformed [15]. The criteria for foraminal qualitative assessment were as follows: grade 0, normal foramina with normal dorsolateral border of the intervertebral disc and normal form of the foraminal epidural fat (oval or inverted pear shape); grade 1, slight foraminal stenosis and deformity of the epidural fat with the remaining fat still completely surrounding the exiting nerve root; grade 2, marked foraminal stenosis and deformity of the epidural fat with the remaining fat only partially surrounding the exiting nerve root; and grade 3, advanced stenosis with obliteration of the epidural fat [15, 16]. The quantitative measures were performed by a trained radiologist with blindness to the treatment of patients.

### DTI protocol

A 3-T conventional MRI scanner (Achiva; Philips, Netherlands) was used in this study. Subjects were scanned in a supine position using an eight-channel phased array spine coil. DTI was performed using an echo-planar imaging sequence with a free breathing scanning technique. The following imaging parameters were set: 0.600 s/mm^2^ *b*-value; MPG, 15 directions (Philips DTI medium); 6000/76 ms for TR/TE, respectively; axial section orientation,3/0-mm section thickness/gap; 200 ×  200 × 160 mm^3^ FOV; 64 × 78 matrix; 3.13 × 2.54 × 3.00-mm^3^ actual voxel size; 1.56 × 1.56 × 3.00 mm^3^ calculated voxel size; NSA, 3; 40 total sections; and 5 min 32 s scan time.

T2-weighted 3D fast field echo sequences were obtained using a 33/3.9 ms for TR/TE; 80 × 80 matrix; FOV 160 × 160 × 200 mm^3^; NSA, 1; gap, 0 mm; 2.00 × 1.99 × 4.00-mm^3^ actual voxel size; and 0.50 × 0.50 × 2.00 mm^3^ calculated voxel size.

### Image analysis

**Fig. 3:**
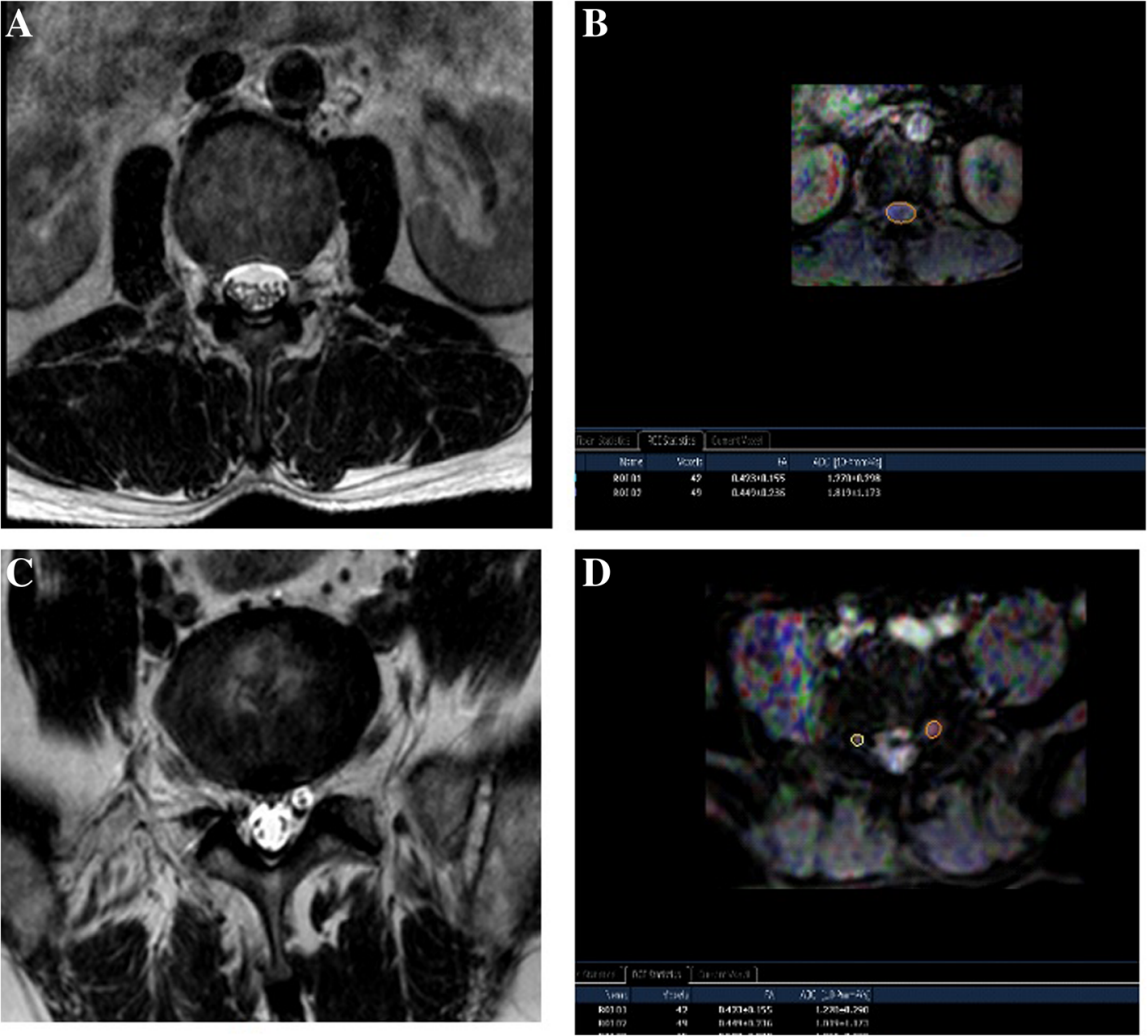
The cauda equina MRI T2W image (**a**) and its FA mapping of DTI (**b**). ROIs were placed on the cauda equina on the zones equally to the disc plane (superior 1/3, middle1/3, and inferior 1/3) on the FA mapping, and the FA values were calculated (**b**), the minimum value of three zones was taken as the cauda equina FA at that level. MRI T2W image of bilateral nerve roots (**c**) and FA mapping of DTI of bilateral nerve roots (**d**). ROIs were placed on the:“intraspinal,” “intraforaminal,” and “extraforaminal” zones of bilateral nerve roots on FA mapping and the FA values were calculated (**d**), the minimum value of three zones was taken as the nerve root FA value. MRI, magnetic resonance imaging; FA, fractional anisotropy; DTI, diffusion tensor imaging

After DTI data were transferred to a PC, a Philips Extended Workspace (Philips DICOM Viewer R2.6 SP1) was used. Using the fiber tracking application software, the anatomical images were superimposed on a fractional anisotropy (FA) map to permit anatomical correlation (Fig. 3). The diffusion tensor was calculated using a log-linear fitting method. On axial images, the regions of interest (ROIs) were placed at cauda equina and the nerve roots of a level freehand, to circumscribe cauda equina or nerve roots with minimal inclusion of cerebral spinal fluid (CSF). In the cauda equina, ROIs were placed on the zones the same as the disc, including superior 1/3, middle1/3, and inferior 1/3 of the disc, taking the minimum value of three zones as the FA value of the cauda equina. In lumbar spinal nerves, ROIs were placed on the “intraspinal,” “intraforaminal,” and “extraforaminal” zones, taking the minimum value of three zones as the FA value of the nerve root. The FA values were calculated with the software at levels from L1 to S1 in patients. The sizes of ROIs from 25 to 50 mm^2^ and 50 to 150 mm^2^ were selected to be as accurate as possible on the respective nerve roots and the cauda equinas to reduce the partial volume effects when the mean FA value was calculated. All DTI analyses were performed twice by two trained radiologists to avoid intra- and interobserver differences [17], and they were blind to the treatment of patients. The standard was as follows: if a FA value of the lumbar cauda equina or/and the nerve root of the narrow level decreased ≥ 0.1 than that of the non-stenotic or the normal level (commonly took the T12-L1 cauda equina and nerve root values as the reference), it was meaningful and the level should be treated surgically.

### Determining decompression levels

#### Determined by conventional MRI

The levels of central tube are ≤ 76mm^2^ or/and foramen or/and lateral recess ≥ grade 1 narrow.

#### Determined by conventional MRI + NE

The levels that had the features of central tube ≤ 76mm^2^ or/and foramen or/and lateral recess ≥ grade 1 narrow determined by conventional MRI and being located by NE in term of the American Association of Spinal Cord Injury (ASIA). If NE could not locate the level, the levels were determined only by conventional MRI.

#### Determined by conventional MRI + (PM or DTI)

Based on the central tube ≤ 76mm^2^ or/and foramen or/and lateral recess ≥ grade 1 narrow, if the score of PM and/or the FA value of DTI was positive, the level was considered for surgical decompression; if the score of PM and the FA value of DTI were both negative, the level was considered for surgery determined only by conventional MRI. If there were conflicts of the opinion, the two direct of spine surgeons reached a mutual decision through a mutual discussion.

### Outcomes

The primary outcomes were the averages of reference FA value, positive, negative FA value, and positive, negative PM score as well as the levels. The reference FA value often refers to the FA value of L1 the positive and the negative FA value refers to the FA values of positive and negative level, respectively, each level including the cauda equina and the nerve roots of two sides. The positive, negative PM score refers to the PM scores of positive and negative level, each level including the nerve roots of two sides. The levels of decompression were determined by conventional MRI + (PM or DTI), conventional MRI + NE, and reduced levels (the difference between conventional MRI + (PM or DTI) and conventional MRI + NE) in each group.

The secondary outcomes were the visual analog scale pain scores for both back and leg systems (VAS-BP, VAS-LP) and the Oswestry Disability Index (ODI), both have been used frequently in studies involving patients with lumbar spinal stenosis on a scale of 0 to100 [18]. All patients were blind to the roles of pain scores and ODI.

### Assessments

The primary outcomes were assessed at the preoperative. The secondary outcomes were assessed at the preoperative and 2 weeks, 3 months, 6 months, and 12 months after surgeries. Postoperative assessments were used to capture the trajectory and stability of the treatment responses. Institutional ethics review board approval was obtained before commencing the collection of data.

### Statistical analysis

All the measurement values were expressed as a mean ± standard deviation. The primary analysis was implemented with an analysis of the covariance with reduced levels between each group and VAS-BP, VAS-LP, and ODI (the preoperative and the 2 weeks postoperative) in every group. *T* test analysis was undertaken to compare the reduced levels between each group, and VAS-BP, VAS-LP, and ODI (the preoperative and the 2 weeks postoperative) in every group. All statistical analysis was done using IBM SPSS version 19.

## Results

### Characteristics of the study population

**Fig. 4:**
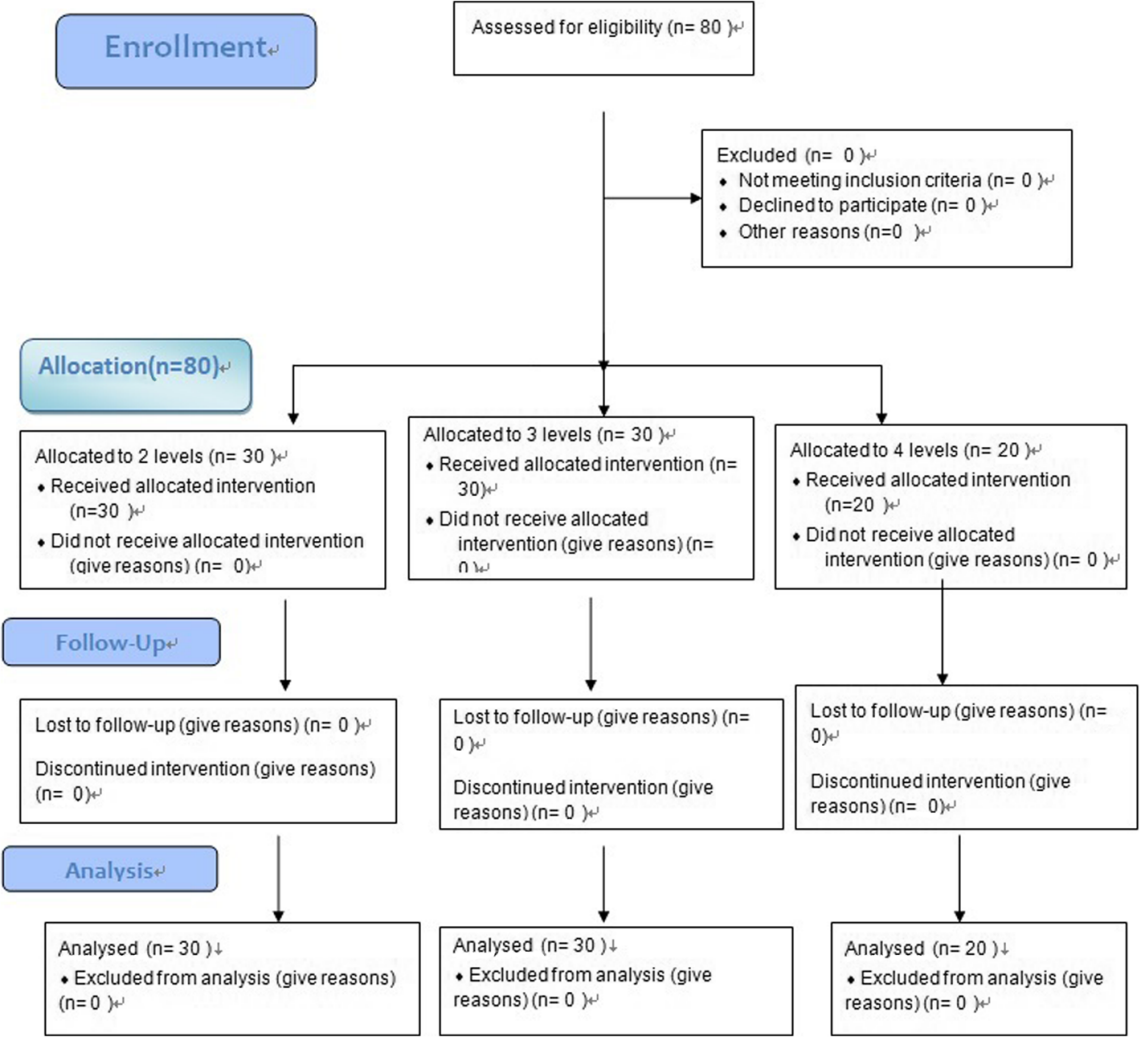
Patient enrollment flow chart

From October 2015 to October 2017, a total of 80 patients (30 two levels, 30 three levels, 20 four levels) (Fig. 4) with degenerative lumbar spinal stenosis detected on conventional MRI were eligible.

### Outcomes

As shown in Table 1, the reference FA values were taken by L1 levels in 76 patients, while in 4 patients, they were replaced by L2 levels because of the L1 levels were narrow. The averages of the reference FA values and the levels were cauda equina, 0.434 ± 0.029 (80); left nerve root, 0.468 ± 0.030 (80); and right nerve root, 0.474 ± 0.023 (80). The averages of the FA values of positive levels and the levels were cauda equina, 0.302 ± 0.028 (134); left nerve root, 0.317 ± 0.025 (25); and right nerve root, 0.317 ± 0.029 (26). The averages of the FA values of negative levels and the levels were cauda equina, 0.428 ± 0.038(93); left nerve root, 0.472 ± 0.043 (93); and right nerve root, 0.489 ± 0.047 (93). No statistically significant difference was found between two radiologists about the FA values. The averages of the PM scores of positive levels and the levels were left nerve root, 3.22 ± 1.33 (77) and right nerve root, 3.01 ± 1.24 (75). The averages of the PM scores of negative levels and the levels were left nerve root, 1 (39) and right nerve root, 1 (39) (Table 2).

**Table 1:** Averages FA values of levels (all levels were considered to be surgical decompression determined only by conventional MRI) and level number

Reference FA values^a^ (*n*)			Positive^c^ FA values (*n*)			Negative FA values^b^ (*n*)		
Cauda equinas (*n*)	Left nerve roots (*n*)	Right nerve roots (*n*)	Cauda equinas (*n*)	Left nerve roots (*n*)	Right nerve roots (*n*)	Cauda equinas (*n*)	Left nerve roots (*n*)	Right nerve roots (*n*)
0.434 ± 0.029 (80)	0.468 ± 0.030 (80)	0.474 ± 0.023 (80)	0.302 ± 0.028 (134)	0.317 ± 0.025 (25)	0.317 ± 0.029 (26)	0.428 ± 0.038 (93)	0.472 ± 0.043 (93)	0.489 ± 0.047 (93)

*FA* fractional anisotropy

^a^The reference FA values were measured by L1 levels in 76 patients, while in 4 patients, replaced by L2 levels, because of narrow

^b^All the negative FA values were considered only nerve roots and/or cauda equinas in the negative levels

^c^If the narrow level FA value decreased ≥ 0.1 than the reference, the level was positive

**Table 2:** Level number and PM

Project	Positive PM		Negative^a^ PM	
	Left nerve root	Right nerve root	Left nerve root	Right nerve root
Levels (*n*)	77	75	39	39
Scores (average)	3.22 ± 1.33	3.01 ± 1.24	1	1

*PM* paraspinal mapping

^a^PM scores = 1, one level, one side

**Fig. 5:**
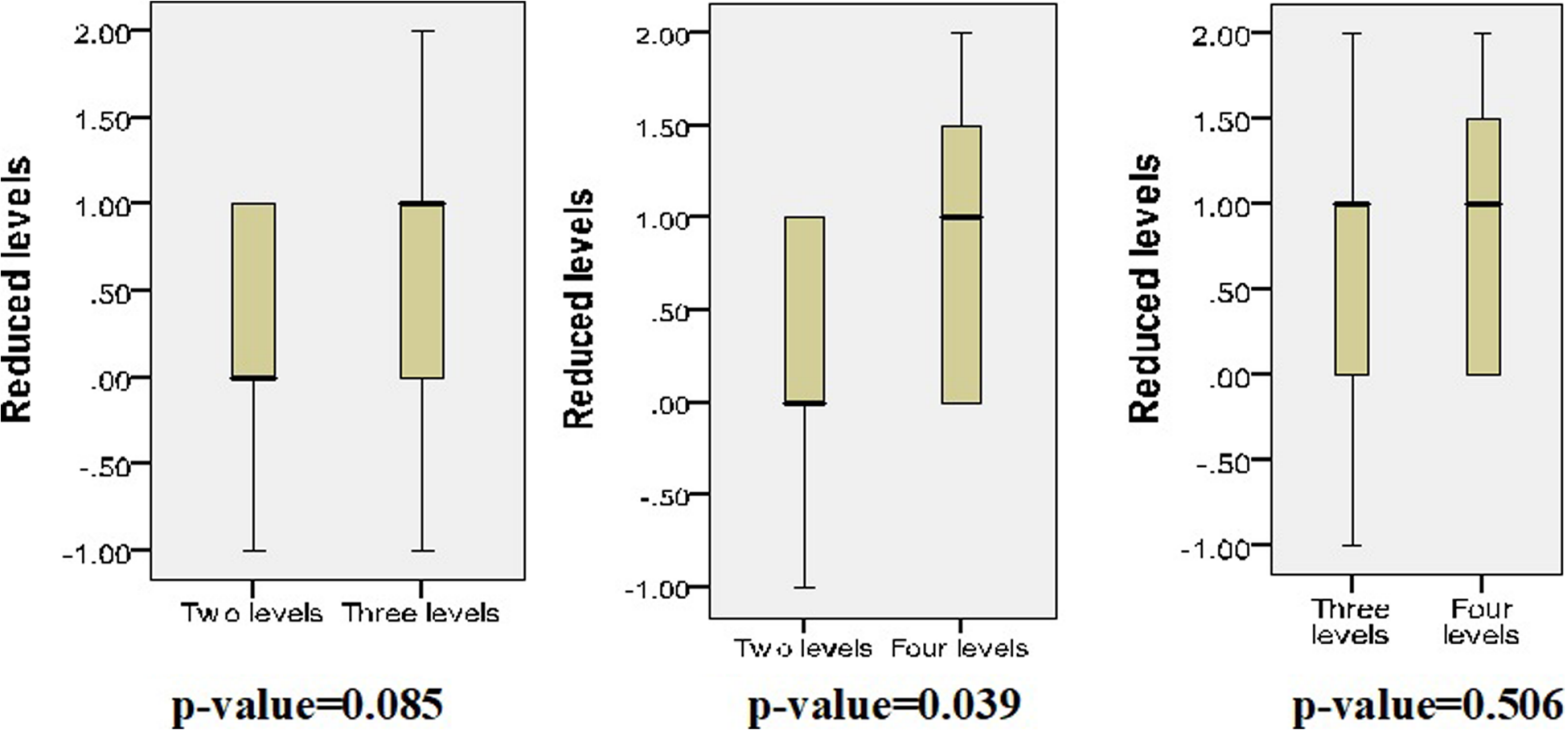
The reduced levels had no statistical significance differences between each other except for two levels and four levels

Tables 3 and 4 and Fig. 5 showed that the reduced levels of every group had no statistical significant differences between each other except for two levels and four levels (two levels/three levels, *p* = 0.085; two levels/four levels, *p* = 0.039; three levels/four levels, *p* = 0.506, respectively). No conflicts of the opinions happened in the decision of decompression levels with all patients.

**Table 3:** Average decompression levels between two methods

Group (*n*)	Determination by MRI + NE	Determination by MRI + (PM or DTI)	*p* value
1 (30)	1.77 ± 0.430	1.400 ± 0.498	0.001
2 (30)	2.67 ± 0.547	2.000 ± 0.455	0.000
3 (20)	3.15 ± 0.813	2.300 ± 0.571	0.000
Total (80)	2.45 ± 0.810	1.850 ± 0.618	0.000

*DTI* diffuse tensor imaging, *PM* paraspinal mapping, *1* two levels, *2* three levels, *3* four levels

**Table 4:** Average reduced levels by using conventional MRI + (PM or DTI) on very group

Surgical characteristics	1 (*n* = 30)	2 (*n* = 30)	3 (*n* = 20)
Reduced levels	0.40 ± 0.56	0.70 ± 0.75	0.85 ± 0.81

*DTI* diffuse tensor imaging, *PM* paraspinal mapping, *1* two levels, *2* three levels, *3* four levels

**Fig. 6:**
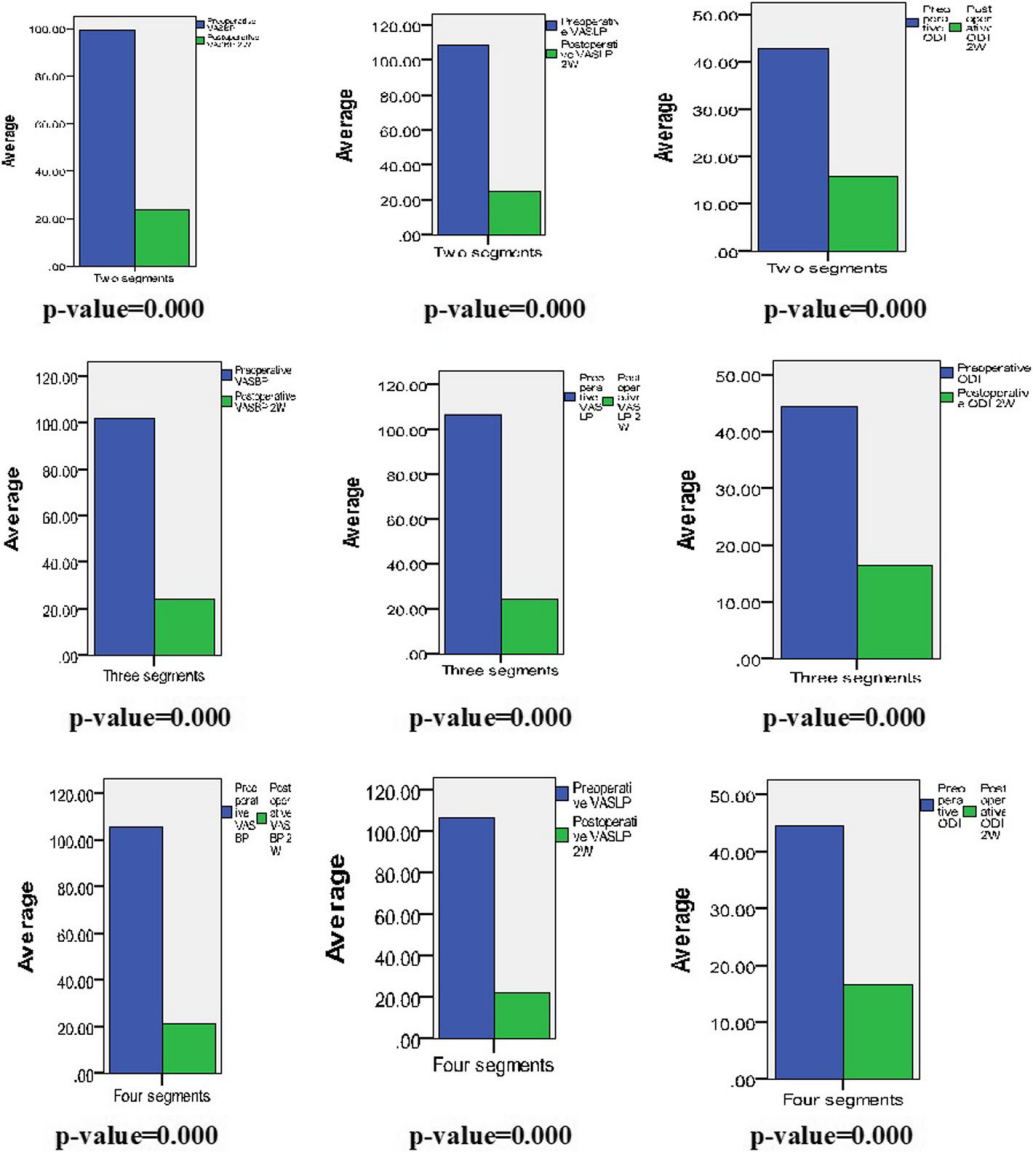
Improvements in functional and pain scores at 2 weeks postoperatively compared to that preoperatively in three groups. ODI, Oswestry Disability Index; VASBP, visual analog scale for back pain; VASLP, visual analog scale for leg pain

All postoperative ODI, VAS-BP, and VAS-LP scores at 2 weeks after surgeries were measured less than preoperative ODI, VAS-BP, and VAS-LP scores with statistically significance and the average of ODI, VAS-BP, and VAS-LP scores were greatly reduced from before surgeries to 2 weeks after surgeries (Fig. 6, Table 5). There were some time order improvement of VAS-BP and VAS-LP scores and ODI scores at 2 weeks, 3 months 6 months, and 12 months in every group postoperative (Table 5).

**Table 5:** Average scores of VAS and ODI

Projects	Preoperative	2 weeks	3 months	6 months	12 months
VAS-BP1	99.43 ± 25.73	23.53 ± 11.26	16.27 ± 3.13	14.07 ± 2.97	10.17 ± 1.51
VAS-LP1	108.80 ± 22.23	24.80 ± 11.31	16.40 ± 3.53	14.10 ± 2.62	8.90 ± 1.60
ODI 1	42.77 ± 9.60	15.97 ± 3.40	13.57 ± 1.65	13.37 ± 1.16	6.30 ± 0.84
VAS-BP2	102.10 ± 19.70	24.27 ± 7.66	17.73 ± 3.41	15.07 ± 3.28	9.70 ± 1.50
VAS-LP2	107.07 ± 20.93	24.67 ± 7.77	16.90 ± 3.67	14.43 ± 3.09	7.80 ± 1.35
ODI 2	44.40 ± 7.04	16.50 ± 2.43	13.70 ± 1.86	11.47 ± 1.41	6.57 ± 0.77
VAS-BP 3	105.55 ± 16.42	21.20 ± 4.58	16.50 ± 3.41	13.05 ± 3.17	8.55 ± 1.73
VAS-LP3	106.75 ± 17.33	22.40 ± 4.68	16.75 ± 3.01	13.20 ± 2.93	7.10 ± 1.02
ODI3	44.65 ± 6.97	16.60 ± 2.11	13.20 ± 1.61	11.25 ± 1.16	5.70 ± 1.03

Data was presented as mean ± SD

*ODI* Oswestry Disability Index, *VASBP* visual analog scale for back pain, *VASLP* visual analog scale for leg pain, *1* two levels, *2* three levels, *3* four levels

## Discussion

In this study, the averages of reference FA values were cauda equina, 0.434 ± 0.029; left nerve root, 0.468 ± 0.030; and right nerve root, 0.474 ± 0.023. The averages of the FA values of negative levels were cauda equina, 0.428 ± 0.038; left nerve root, 0.472 ± 0.043; and right nerve root, 0.489 ± 0.047. Our FA values of nerve roots were not comparable to those obtained in the study of lumbar spinal nerves by Balbi et al. [19] (0.218) and Haakma et al. [20] (0.31) and might be because of the different software calculation methods. Also, our average FA values of the cauda equine were lower than the other reported (0.492) [21], because as our pre-paper [8], at the L1 level, the FA value we measured was actually a FA value of the mixture of gray matter, white matter, and cerebrospinal fluid, and at L2-S1 levels, the FA value we measured was actually a FA value of the mixture of cauda equina nerve and cerebrospinal fluid; the lower FA value of cerebrospinal fluid would reduce the FA value.

Our 3.13 × 2.54 × 3.0 mm^3^ voxel size was very larger than that in the previous study (1.1 × 1.6 × 3 mm), and therefore, the spatial resolution was unlikely to account for the difference [21], maybe because attempt to increase the resolution by decreasing the voxel size would lead to the bad result in lumbar nerve root imaging. As our pre-paper [8], the FA values of the cauda equina were typically lower than the actual values and this might be due in part to the volume averaging with CSF in each voxel. All these affected the FA values of the reference and the narrow level, but no difference was observed.

The reduced surgical levels showed statistically significant difference between two levels/four levels, but it was not significant between two levels/three levels and three levels/four levels, which showed that, with the surgical levels determined by conventional MRI increasing, the benefits of reducing the decompression level of DTI and PM would be uncertainly more obvious.

All postoperative ODI, VAS-BP, and VAS-LP scores at 2 weeks after surgeries were measured to be with improvement than preoperative ODI, VAS-BP, and VAS-LP scores with statistically significance, respectively, and the averages of ODI, VAS-BP, and VAS-LP scores were greatly reduced, indicating that surgical treatment achieved good results. In the postoperative follow-up, there were 4 patients with lumbar compression fractures (because of osteoporosis), 2 patients with femoral neck fractures, which all had nothing to do with the lumbar spinal stenosis. The postoperative VAS-BP and VAS-LP scores and ODI scores in some cases had some fluctuation, but the averages were toward improvement timely, and none of the patients’ symptoms recurred or/and exacerbated or/and required a repeat surgery.

### Limitations

We acknowledged that our study had some limitations. One was that a small number of subjects were investigated and a limited follow-up was performed. The further studies were needed to investigate whether our findings remain valid in a larger population and a longer follow-up. Another, we could not repeat the DTI and PM after surgeries because of the spinal instrumentation artifacts, such as those from pedicle screw systems and surgical scar, respectively.

## Conclusions

This study suggested that with the surgical levels determined by conventional MRI increasing, the benefits of DTI and PM will be uncertainly more obvious.

## Abbreviations

ASIA: American association of spinal cord injury
CSF: Cerebral spinal fluid
DSCSA: Dural sac cross-sectional area
DTI: Diffuse tensor imaging
EMG: Electromyography
FA: Fractional anisotropy
LSS: Lumbar spinal stenosis
MRI: Magnetic resonance imaging
NE: Neurogenic examination
ODI: Oswestry Disability Index
PM: Paraspinal mapping
ROIs: Regions of interest
VAS-BP: Back visual analog scale pain
VAS-LP: Leg visual analog scale pain
